# Quantitative imaging of bone remodeling in patients with a unicompartmental joint unloading knee implant (ATLAS Knee System)—effect of metal artifacts on a SPECT-CT-based quantification

**DOI:** 10.1186/s40658-021-00360-z

**Published:** 2021-02-17

**Authors:** Oliver S. Grosser, Marcus Klutzny, Heiko Wissel, Dennis Kupitz, Michael Finger, Simone Schenke, Jan Wuestemann, Christoph H. Lohmann, Christoph Hoeschen, Maciej Pech, Christian Staerke, Michael C. Kreissl

**Affiliations:** 1 Department of Radiology and Nuclear Medicine, University Hospital Magdeburg and Medical Faculty of Otto-von-Guericke University, Leipziger Strasse 44, 39120 Magdeburg, Germany; 2 Research Campus STIMULATE, Otto-von-Guericke University, Magdeburg, Germany; 3 Department of Orthopaedic Surgery, University Hospital Magdeburg and Medical Faculty of Otto-von-Guericke University, Magdeburg, Germany; 4 Chair of Medical Systems Technology, Institute of Medical Engineering, Faculty of Electrical Engineering and Information Technology, Otto-von-Guericke University, Magdeburg, Germany

**Keywords:** Hybrid SPECT-CT, Knee implant system, Joint unloading implant, Optimization, Bone remodeling

## Abstract

**Background:**

SPECT-CT using radiolabeled phosphonates is considered a standard for assessing bone metabolism (e.g., in patients with osteoarthritis of knee joints). However, SPECT can be influenced by metal artifacts in CT caused by endoprostheses affecting attenuation correction. The current study examined the effects of metal artifacts in CT of a specific endoprosthesis design on quantitative hybrid SPECT-CT imaging.

The implant was positioned inside a phantom homogenously filled with activity (955 MBq ^99m^Tc). CT imaging was performed for different X-ray tube currents (*I* = 10, 40, 125 mA) and table pitches (*p* = 0.562 and 1.375). X-ray tube voltage (*U* = 120 kVp) and primary collimation (16 × 0.625 mm) were kept constant for all scans. The CT reconstruction was performed with five different reconstruction kernels (slice thickness, 1.25 mm and 3.75 mm, each 512 × 512 matrix). Effects from metal artifacts were analyzed for different CT scans and reconstruction protocols. ROI analysis of CT and SPECT data was performed for two slice positions/volumes representing the typical locations for target structures relative to the prosthesis (e.g., femur and tibia). A reference region (homogenous activity concentration without influence from metal artifacts) was analyzed for comparison.

**Results:**

Significant effects caused by CT metal artifacts on attenuation-corrected SPECT were observed for the different slice positions, reconstructed slice thicknesses of CT data, and pitch and CT-reconstruction kernels used (all, *p* < 0.0001). Based on the optimization, a set of three protocols was identified minimizing the effect of CT metal artifacts on SPECT data. Regarding the reference region, the activity concentration in the anatomically correlated volume was underestimated by 8.9–10.1%. A slight inhomogeneity of the reconstructed activity concentration was detected inside the regions with a median up to 0.81% (*p* < 0.0001). Using an X-ray tube current of 40 mA showed the best result, balancing quantification and CT exposure.

**Conclusion:**

The results of this study demonstrate the need for the evaluation of SPECT-CT protocols in prosthesis imaging. Phantom experiments demonstrated the possibility for quantitative SPECT-CT of bone turnover in a specific prosthesis design. Meanwhile, a systematic bias caused by metal implants on quantitative SPECT data has to be considered.

**Supplementary Information:**

The online version contains supplementary material available at 10.1186/s40658-021-00360-z.

## Background

In recent years, an increasing number of total knee and hip arthroplasties have been observed [1, 2]. Data from the OECD (Organization for Economic Co-operation and Development) countries show that a mean of 166.4 hip and 126.2 knee replacements per 100,000 inhabitants and year are performed (data from the USA: 225.8 hip and 203.5 knee replacements per 100,000 inhabitants and year) [1]. Today, partial or total knee replacement represents the gold standard in the treatment of end-stage osteoarthropathy of the knee [3–5]. Recently, an extraarticular implant preserving the joint became available for minimally invasive treatment in patients with mild-to-moderate medial osteoarthritis (OA) of the knee by unloading the medial compartment of the joint [6, 7]. The device is used in patients who are not yet candidates for total joint replacement surgery (e.g., due to age, activity level or resistance to invasive, irreversible surgeries). The extraarticular implant provides the opportunity for an intermediate treatment between conservative care and joint sacrificing surgery to reduce the treatment gap in knee OA patients [8].

In addition, the availability of specialized or advanced prosthetic designs in combination with innovative composite materials is increasing [7, 9–13]. In this situation, specific knowledge regarding bone remodeling (e.g., time of response and intensity of bone turnover) was demonstrated to be essential for optimization of patient therapy/physical therapy. This especially holds true when new prosthetic designs or implant systems are introduced [9, 10, 14].

The use of SPECT-CT methodology with dedicated radiopharmaceuticals is established in imaging bone metabolism [15, 16] and postoperative management after prosthetic implantation [16, 17]. However, CT metal artifacts can impair SPECT image quality and SPECT-based quantitation because of CT-based attenuation correction [17–19]. Artifacts, e.g., artificial modulations in HU values, reflect the typical composition and geometry of an implant and cannot be generalized. Therefore, evaluation is necessary for each implant. Currently, results are available for some specific designs [10, 17, 20]. In parallel with the development of new prostheses (e.g., new composite materials and geometries) and the corresponding clinical procedure (e.g., implantation), optimization of nuclear medicine imaging procedures is also essential. Therefore, SPECT-CT imaging of bone turnover in patients with prostheses (or in our setup, a unicompartmental load absorbing knee implant [6, 7]) using a dedicated radiotracer has also to be tested for the influence of metal artifacts on attenuation correction of SPECT data reconstruction and quantitation of bone metabolism.

In the present phantom study, we investigated the effect of the abovementioned, novel unicompartmental implantable joint-unloading prosthesis for the treatment of medial knee OA on quantitative SPECT data. We analyzed the potential to minimize the error introduced by CT-based attenuation correction on reconstructed SPECT data by using different CT protocols. Additionally, radiobiological optimization of low-dose CT (LD-CT) imaging, preserving an acceptable level of quantitative data while reducing the radiation dose as much as possible, was performed.

## Methods

### Joint unloading system and phantom setup

**Fig. 1:**
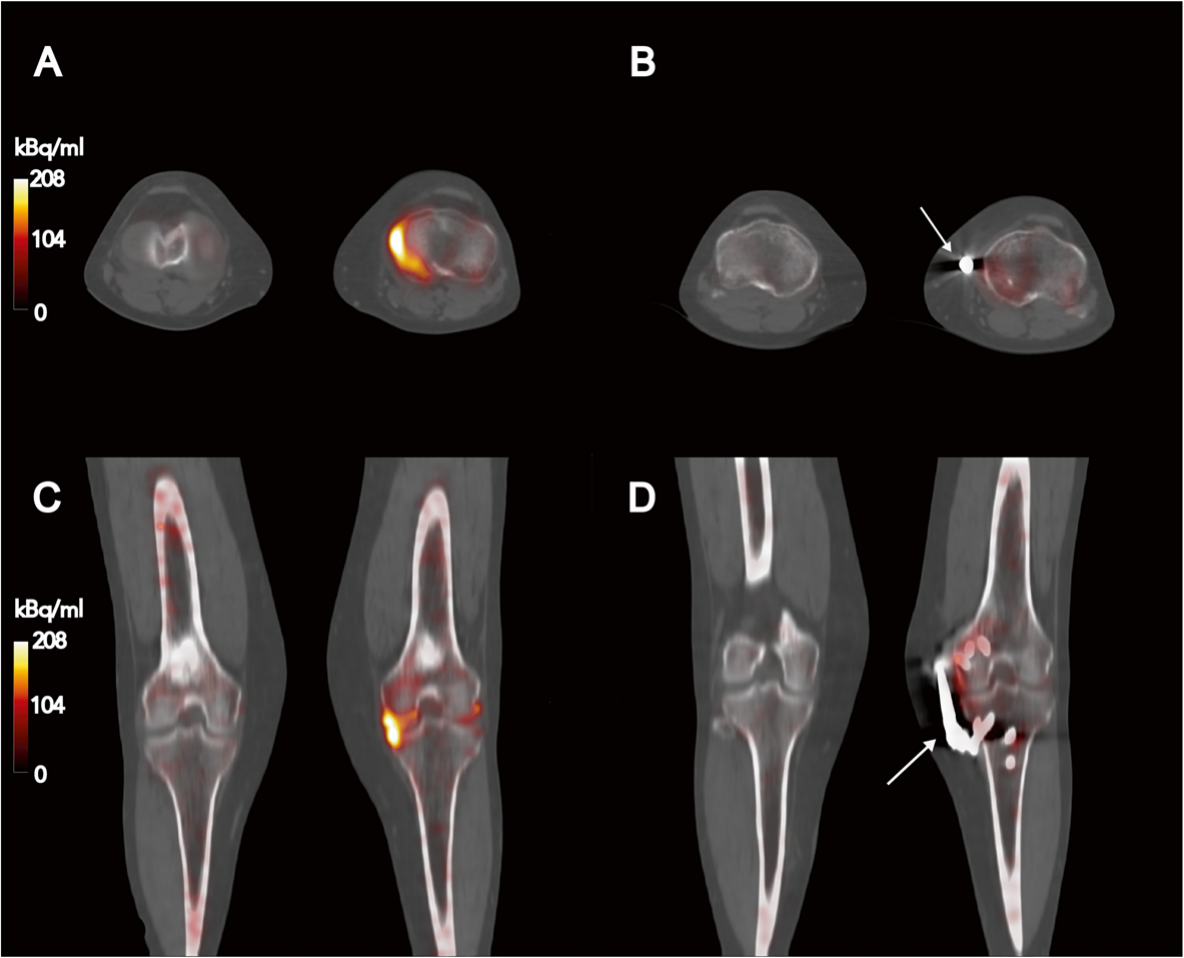
**a**, **c** Pre-OP SPECT-CT images from a patient (f, 46 year, SPECT-CT: 5 months pre-implantation) with beginning knee OA and **b**, **d** corresponding post-op imaging (14 months post-implantation) exemplifying the effect from the examined load absorber (**b**, **d**: metal artifact labeled by arrow). Patient showing a significant decrease in bone turnover after implantation at the level of the left tibia plateau (AC_max, pre_ = 309.2 kBq/ml, [SUV_max_ = 77.5] and AC_max, post_ = 99.0 kBq/ml, [SUV_max_ = 31.7]). Accumulation patterns from bone turnover (pre/post) were windowed identically

**Fig. 2:**
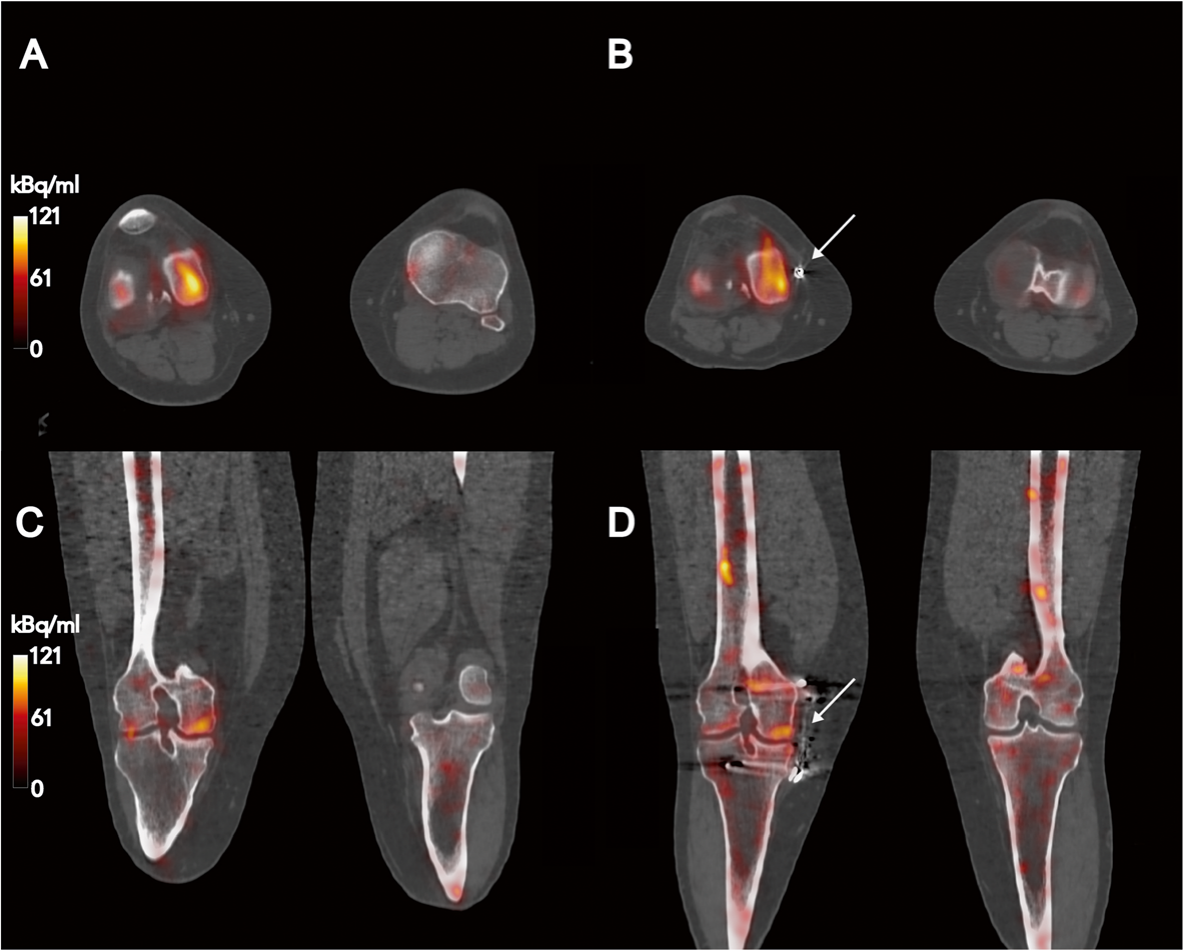
**a**, **c** Pre-op SPECT-CT images from a patient (f, 36 year, SPECT-CT: 8 months pre-implantation) with a secondary gonarthrosis and **b**, **d** corresponding post-op imaging (10 months post-implantation) exemplifying the effect from the examined knee implant system (**b**, **d**: metal artifact labeled by arrow). Patient showing a constantly increased uptake at the medial condyle of the right femur after implantation of the load absorber (AC_max, pre_ = 118.6 kBq/ml [SUV_max_ = 35.6] and AC_max, post_ = 110.89 kBq/ml, [SUV_max_ = 32.9]). Accumulation patterns from bone turnover (pre/post) were windowed identically

The analyzed load absorber (Atlas Knee System®, Moximed Inc., Hayward, CA, USA) was designed to reduce stress on the medial knee compartment in patients with OA. The specific effect of the system was assessed by (pre-/post-implantation) SPECT-CT imaging for two typical accumulation patterns. The specific effects (accumulation patterns) will be exemplified by two cases (Figs. 1 and 2). In one patient, a significant reduction in bone metabolism (Fig. 1) was demonstrated after insertion of the implant. In contrast, only a slight decrease in bone turnover was detected by SPECT-CT in the second patient (Fig. 2).

**Fig. 3:**
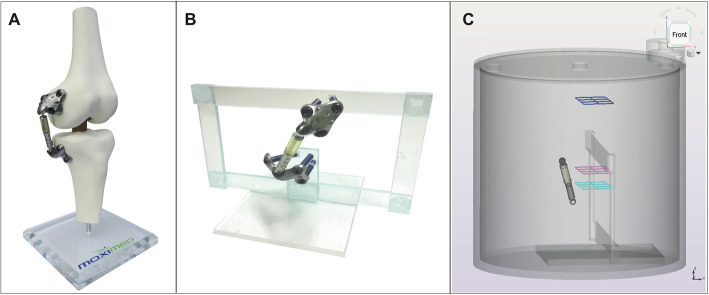
Atlas® Knee System **a** mounted to a model knee joint, **b** mounted to the acryl frame used for positioning inside the cylindric phantom, and **c** technical drawing of acryl frame with load absorber inserted in the phantom (analyzed ROI positions are visualized, turquoise: lateral tibia head, magenta: femur head, dark blue: reference region). The view on the phantom (CAD-drawing) is from an anterior lateral position (plates and screws of the load absorber were not visualized)

The load absorber is mounted outside and medial to the knee joint (Fig. 3a). It is placed under the skin without bone, cartilage, or ligament resection; it contains a dedicated absorber positioned between femoral and tibial bases on the medial side of the knee. The device is composed of a set of materials (titan alloy: self-tapping locking screws, tibial and femoral bases; the shock absorber is made from cobalt-chromium steel and polycarbonate urethane) covering a wide range of density values and, therefore, the attenuation of X-rays.

The effect of the implant system on hybrid SPECT-CT image quality (defined as the achievable accuracy of quantification and the homogeneity of reconstructed activity concentration) in the knee joint was examined by using a dedicated phantom setup (Fig. 3b). The unloading device was mounted to a thin acryl frame (thickness = 4 mm) and placed in a standard cylindric SPECT phantom without inserts, such as spheres (Flangeless Deluxe Jaszczak Phantom, Data Spectrum, Durham, NY, USA). The wide-open frame was chosen to prevent artificial effects from material/inhomogeneities in activity concentration and density in the examined region (Fig. 3b). For imaging, the phantom was filled with a water-based solution with a defined activity concentration (see section SPECT-CT imaging). For discussion on the dimension of the phantom, see also Supplementary Data (Section 1).

### CT imaging in hybrid SPECT-CT

All examinations were performed using a dedicated hybrid SPECT-CT (Discovery NM/CT 670, GE Healthcare, Milwaukee, USA). The integrated CT component is identical to a 16-slice CT used in diagnostic CT imaging (model: Bright Speed 16, GE Healthcare, Milwaukee, USA). CT scans of the phantom were performed in an axial field-of-view (FOV) with a diameter of 50 cm by helical scans with a gantry rotation time *t*_rot_ = 0.8 s and a primary collimation of 16 × 0.625 mm. CT imaging of the phantom setup was performed with different scan protocols varying the X-ray tube current (*I* = 10, 40, 125 mA) and the table pitch (*p* = 0.562 and 1.375). The X-ray tube voltage (*U* = 120 kVp) and the primary collimation of 16 × 0.625 mm were kept constant for all protocols. Each combination of X-ray tube current and pitch was used for imaging of the identical phantom geometry. The LD-CT scans were performed without an angular variation of the X-ray tube current.

The CT reconstruction was performed with a slice thickness of 1.25 mm and 3.75 mm (each 512 × 512 matrix, pixel size 0.977 × 0.977 mm^2^) and with five different manufacturer prespecified reconstruction kernels each (“Standard plus,” “Bone Plus,” “Bone Plus and IQE,” “Bone+ Plus,” “Bone+ Plus and IQE”) (Table 1, [22]) for each performed CT scan, if applicable. The system provided image reconstruction using an extended HU-scale to avoid typical truncation artifacts from metal implants usually observed in standard 12-bit HU scale [−1024HU, +3071HU]. A dedicated reconstruction algorithm for reduction of the metal artifacts was not available for the CT used.

**Table 1:** Analyzed CT reconstruction setups

Protocol ID	Name of protocol^**a**^	Description
S Plus	Standard Plus	Standard CT reconstruction kernel used for routine exams (e.g., chest and abdomen in diagnostic applications), also recommended for reconstruction of LD-CT data in hybrid SPECT-CT imaging. Additional “Plus” option.
B Plus	Bone Plus	Bone reconstruction kernel an activated “Plus” option.
B Plus IQE^b^	Bone Plus with IQE	Bone reconstruction kernel, activated “Plus” option and IQE mode
B+ Plus	Bone+ Plus	Bone+ reconstruction kernel and activated “Plus” option
B+ Plus IQE^b^	Bone+ Plus with IQE	Bone+ reconstruction kernel, activated “Plus” option, and IQE mode.

*IQE* Image quality enhancement mode: including compensation for helical artifacts, IQE is not available for reconstructions with a slicing of 3.75 mm

“Plus” mode: reconstruction with additional views of helical scanned data from an up to 20% increased slice profile

^a^CT reconstruction kernel and options (e.g., reduction of artifacts from helical scanning) corresponding to manufacturer-specific product name

^b^For further details, see Solomon et al. [21]

### SPECT-CT imaging and analysis of data

SPECT data were obtained for the phantom with the load absorber. The phantom was homogeneously filled with 955 MBq of ^99m^Tc-pertechnetate (decay corrected to the start of the SPECT acquisition) diluted in water (background volume = 5812 ml, activity concentration 164.3 kBq/ml). Projection data were measured over 360° (energy window 141 keV ± 10%, 60 projections at steps of 6°, and 30 s/projection) with a 256 × 256 matrix (pixel size = 2.21 × 2.21 mm^2^, zoom = 1.0) for a single bed position. A separate scatter window was measured at 120 keV ± 5%. The phantom-to-detector distance was minimized by the real-time automatic body contouring of the gamma camera. A SPECT imaging protocol and a quantitative reconstruction setup of the SPECT data (3D-OSEM: 3D-Ordered Subset Expectation Maximization with 4 iterations and 10 subsets, no additional pre-/post-filtering) were chosen in accordance with the basic scan protocol for SPECT-CT imaging in routine clinical practice. CT-based attenuation correction (CTAC) of the emission data from SPECT imaging was executed by using the previously described set of LD-CT images. SPECT reconstruction was performed for each specified CT reconstruction (defined by variations of X-ray tube current, pitch, slice thickness, and CT reconstruction kernel). Processing was carried out on a dedicated workstation (Xeleris 4, GE Healthcare, Milwaukee, USA) with a specific algorithm for quantitative image reconstruction (“Preparation for Q.Metrix” software application, GE Medical, Milwaukee, USA), including resolution recovery, scatter correction (scatter fraction factor [SCF] = 1.1), attenuation correction by μ-maps estimated from LD-CT [23], and system sensitivity of the SPECT detectors.

**Fig. 4:**
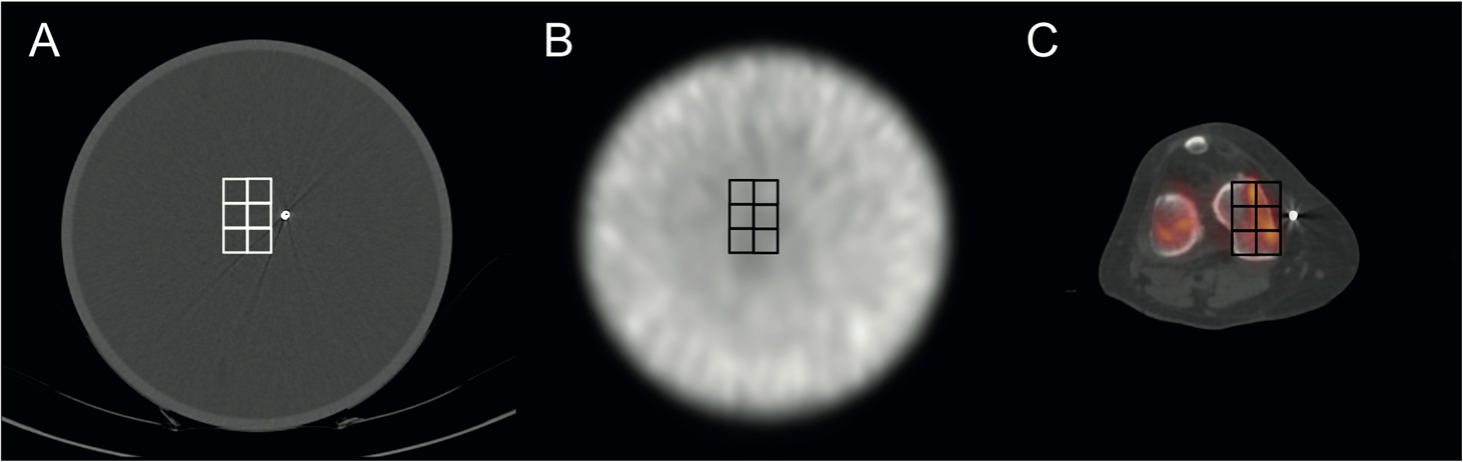
Positioning of the standardized ROI set in the hybrid phantom (femur region) in correlation to the load absorber. **a** Positioning of the ROI set in the CT scan for analyzing the effect of the CT artifact from the joint-unloading prosthesis on HU values and **b** the corresponding attenuation corrected quantitative SPECT data. **c** Identic ROI setup transferred to a typical patient illustrating the correlation between the ROI set and the examined anatomic region (exemplified the femur region)

The LD-CT, the μ map, and the quantitative SPECT images were analyzed for all voxels in a set of regions of interest (ROIs, in total 6 ROIs, e.g., 7 × 7 pixel for SPECT and μ map, CT in concordance to modality-specific resolution), which were defined in addition to the implanted system at the presumed position of the knee joint (Figs. 3c and 4). The analysis was performed for three slices correlating to a typical axial slice of the tibia and the femur condyle. Additionally, a reference volume with a homogeneous density/activity concentration, defining the standard (HU, μ-map, reconstructed activity concentration) without any artifact from the load absorber, was evaluated using the same set of ROIs. ROI analysis was performed by ImageJ V1.52a [24].

### Statistics

The R software package (version 4.02; R Foundation for Statistical Computing) was used for statistical evaluations [25]. CT Hounsfield unit (HU) values, the attenuation coefficients μ within the μ-maps, and the reconstructed activity concentrations within the SPECT images were analyzed for each protocol, defined by the combination of the CT scan protocol and reconstruction setup. Descriptive parameters were expressed as the mean ± standard deviation or median, interquartile range (IQR), and range, if appropriate. All data were tested for normality by the Kolmogorov-Smirnov test. In the case of a non-normal distribution of one of the parameters, the differences in reconstructed HU values, attenuation coefficients (μ map), and activity concentration were tested for significance by the non-parametric Kruskal-Wallis rank-sum test. Pairwise comparison was performed by the Wilcoxon rank-sum test with the Bonferroni–Holm correction applied for multiple comparisons. All tests performed were two-sided, and significance was assumed at a *p* value of less than 0.05.

## Results

### CT imaging

**Fig. 5:**
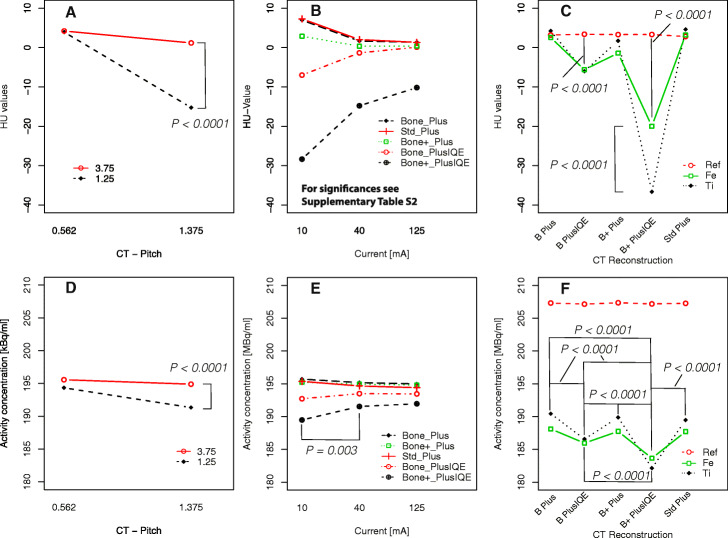
Interaction plots illustrating the effect from **a**, **d** pitch and reconstructed slice thickness, **b**, **e** X-ray tube current and CT-reconstruction/filter setup, **c**, **f** analyzed region and CT-reconstruction/filter setup. Effects are shown for **a**‑**c** HU values and **d**‑**f** corresponding reconstructed activity concentrations

Overall protocols assessed, the HU values in the analyzed ROI sets were not normally distributed (*p* < 0.0001). A significant effect of the X-ray tube current, slice position, reconstructed slice thickness, pitch, and reconstruction kernel (all, p ≤ 0.0001) on the reconstructed HU values was observed. In general, the observed effects were confirmed by pairwise testing (*p* ≤ 0.0001). Significant effects are visualized by interaction plots (Fig. 5a‑c). Distribution of CT data (HU values) and SPECT data (reconstructed activity concentration) including corresponding results from statistical analysis were provided (Supplementary Data, Section 2, Table S1-S6). A significant effect of the X-ray tube current on the HU values was observed for all reconstructions comparing CT data from scans with I = 10 mA and 40 mA (*p* ≤ 0.02, Fig. 5b and Supplementary Data Section 2, Table S2). A difference in HU values between 40 mA and 125 mA was observed for CT data reconstructed by “B+ Plus IQE” and “S Plus” (*p* ≤ 0.001). Moreover, no significant difference was detected in the pairwise comparison of HU values reconstructed with kernels “S Plus” and “B Plus” (*p* = 0.12, Fig. 5b). All other combinations of reconstructions (reconstruction kernels) showed a significant difference in HU values (all *p* < 0.0001, Fig. 5b). Analyzing the individual difference in HU value distribution between the “anatomical” level’s femur and tibia, a significant effect was observed for reconstruction “B+ Plus IQE” (Fig. 5c and Supplementary Data, Section 2, Table S3). There was no significant difference in HU values between the reference slice and both “anatomical” levels for the other CT reconstruction setups investigated (*p* < 0.0001).

**Fig. 6:**
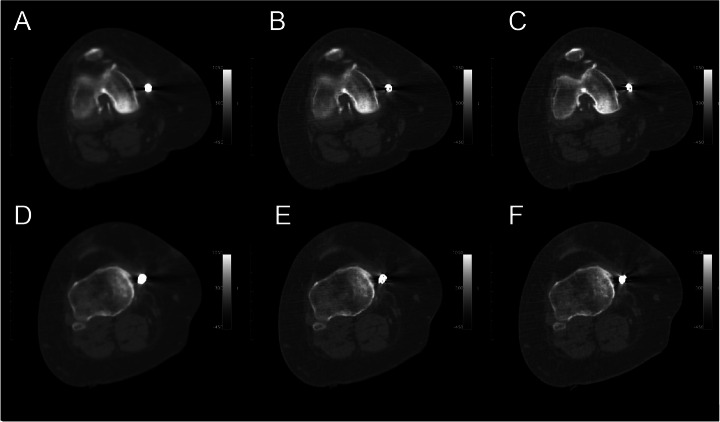
Low-dose CT scan of a female patient (48 year) with an Atlas® Knee System in the right joint. Multiple reconstructions from the same CT detector raw data (120 kV, 55 mA, *t*_rot_ = 0.8 s, pitch = 1.375). Reconstruction is shown for the region of femur (**a**‑**c**) and the tibia (**d**‑**f**). CT reconstruction was performed with setup **a**, **d** “S Plus” (slicing = 3.75 mm), **b**, **e** “B+ Plus” (slicing = 3.75 mm), and **c**, **e** “Bone Plus IQE” (slicing 1.25 mm)

The specific effect of the CT reconstruction on the LD-CT image was exemplified for a patient using a set of CT scan reconstructions for the identical raw data set (Fig. 6).

### Attenuation maps/μ maps

The effects observed for CT data were propagated to the derived μ map. Data were not normally distributed (*p* < 0.0001). The X-ray-tube current, slice position, reconstructed slice thickness, pitch, and CT reconstruction had a significant effect on the estimated μ value (all *p* ≤ 0.0001).

### SPECT imaging

The quantitative SPECT data (reconstructed activity concentration) were also not normally distributed (*p* < 0.0001). In contrast to the HU and μ values, significant effects were only observed for variations of the reconstructed slice thickness and pitch (*p* < 0.0001, Fig. 5d, Table S4), CT reconstruction used for attenuation correction of SPECT data (*p* < 0.0001, Fig. 5e, Table S5), and slice position (femur, tibia, and reference slice, *p* < 0.0001, Fig. 5f, Table S6). A significant effect of the X-ray-tube current on the reconstructed activity concentration was only observed if CT data from “B+ Plus IQE” reconstruction was used for attenuation correction of SPECT data (*p* ≤ 0.003, Fig. 5d and Supplementary Data, Section 2, Table S5).

Moreover, no significant difference was detected by pairwise comparison of quantitative SPECT values reconstructed by using attenuation information from CT reconstructions using kernel “S Plus,” “B Plus,” and “B+ Plus” (all, *p* ≥ 0.29, Fig. 5e). All other combinations of reconstructions (reconstruction setups “B Plus IQE” and “B+ Plus IQE”) showed a significant difference in reconstructed SPECT values (all, *p* < 0.0001, Fig. 5e).

Furthermore, the effect of the different CT reconstruction setups on SPECT data was exemplified for a single patient (Figure S1 and Supplementary Data, Section 3) using several reconstruction algorithms on one CT raw data set (Fig. 6). The individual tracer accumulation was evaluated regarding the uptake pattern in the knee, potentially influenced by the metal artifact from the metal implant, and a reference region (femur shaft) about 10 cm above the knee joint (Figure S1, Supplementary Data). A significant difference was identified in the SPECT data corrected for attenuation by CT from “B+ Plus IQE” setup (*p* < = 0.044, Supplementary Data, Table S7 and Figure S2A). The activity concentration was reduced by a median of about 16‑18%. In the specific case, the reconstructed activity concentration with “B Plus IQE” was slightly reduced but without significance. In contrast, no significant effect by CT reconstruction setup on the measured activity concentration was observed for the reference region (*p* = 1, Supplementary Data, Figure S2B).

### Radiobiological optimization

Taking into account the effects introduced by the hybrid scan and reconstruction protocol on the reconstructed activity concentration (see the results from the preceding analysis), a radiobiological optimized subset (preserving SPECT quantitation while reducing CT exposure) was tested. Subgroup analysis was performed for data acquired with a pitch of 1.375 and X-ray tube currents of 10 and 40 mA. Both parameters were chosen to provide a reduced CT exposure in comparison to other alternatives (10 and 40 mA vs. 125 mA, pitch 1.375 vs. 0.562). Furthermore, CT reconstruction setups showing the smallest deviation between the reference and anatomically assigned slice (femur and tibia) were analyzed (“S Plus,” “B Plus,” and “B+ Plus,” Fig. 5c and f).

In this subset of measurements, the effect of the X-ray tube current on the reconstructed activity concentration was also not significant for the anatomically oriented ROIs (*p* = 0.35). In contrast, a significant effect of the X-ray tube current was detected for the reference region. Compared to data corrected by an LD-CT scan with 125 mA, the activity concentration was overestimated by 1.0% when using LD-CT scans with an X-ray tube current of 10 mA for attenuation (*p* < 0.0001). No significant effect of the attenuation correction on the reconstructed activity concentration was detected by using data from CT scans with 40 mA in comparison to 125 mA.

A significant effect on the reconstructed activity concentration was not observed with respect to the CT reconstruction procedure (“B+ Plus” vs. “S Plus,” *p* = 0.07; all other combinations, *p* ≥ 0.29). The reconstructed quantitative data showed a significant effect depending on the ROI position in the different slices (e.g., femur and tibia, *p* ≤ 0.0001). The median deviation of the reconstructed activity concentration in one slice, representing a surrogate for inhomogeneity, is shown in Table 2. In comparison to the reference region, the activity concentration was significantly underestimated (all, *p* < 0.0001, Table 2). Depending on the CT reconstruction algorithm and the anatomical region, compared to the reference region, the activity concentration inside the ROIs representing the knee joint was estimated to be lower (median difference between −10.1% and −8.9%, Table 2).

**Table 2:** Reconstructed activity concentration, inhomogeneity of reconstructed activity concentration in the anatomically oriented ROIs, and median deviation from the reference region for the optimized scan protocol (40 mA, pitch = 1.375, slicing = 3.75 mm)

Protocol ID	Region	SPECT [kBq/ml]	***P***^***a***^	Inhomogeneity in the slices^**b**^ [%]	***P***^***c***^	Median deviation from reference [%]	***P***^***d***^
S Plus	Femur	186 (183/191) 167‑220	0.019	0.27 (0.00/0.54) −3.23‑7.53	< 0.0001	−10.08 (−11.53/−7.66) -19.27‑6.36	< 0.0001
	Tibia	189 (182/198) 168‑222		0.26 (−3.05/3.18) −4.51‑6.63	< 0.0001	−8.87 (−12.02/−4.28) −18.78‑7.32	< 0.0001
B Plus	Femur	186 (182/191) 166‑220	0.015	0.81 (0.00/0.54) −3.76‑7.53	< 0.0001	−10.13 (−12.06/−7.71) -19.79‑6.30	< 0.0001
	Tibia	188 (182/198) 168‑222		−0.26 (−3.05/3.18) −5.04‑6.63	< 0.0001	−9.16 (−12.06/−4.33) −18.83‑7.26	< 0.0001
B+ Plus	Femur	186 (182/191) 164‑220	0.049	0.00 (−0.40/0.40) −4.30‑7.53	< 0.0001	−10.13 (−12.06/−7.83) −20.76‑6.23	< 0.0001
	Tibia	187 (181/197) 165‑222		0.27 (−2.81/3.74) −5.35‑7.47	< 0.0001	−9.65 (−12.54/−4.81) −20.28‑7.27	< 0.0001

^a^Significance of difference between femur and tibia for the identical reconstruction protocol

^b^Scattering of the observed median in the six different ROIs (*n* = 6) normalized to the median reconstructed activity concentration estimated over all ROIs in the specific slice (anatomical region)

^c^Significant difference between ROIs in the same slice for the identical reconstruction protocol

^d^Significance of the difference between reconstructed activity concentration in the anatomical region and reconstructed activity concentration in the reference region

The preferred LD-CT protocol (40 mA and pitch 1.375) corresponds to a dose length product (DLP) of 137 mGy*cm, corresponding to an effective dose of 0.13 mSv for scanning the full SPECT-FOV [26].

## Discussion

This study examined the propagation of metal artifacts in CT on quantitative evaluation of SPECT data caused by the influence of the CT-based attenuation correction for a specific endoprosthesis design. The potential for optimizing LD-CT scan parameters and parametrization of LD-CT reconstruction was analyzed for this hybrid application. Quantitative effects were examined by phantom measurements with the unloading implant placed inside homogeneous media.

The analysis was performed in ROIs assigned to specific anatomical structures (positions typical for subchondral bone of the femur condyle and the head of the tibia) to analyze the effects of the load absorber on quantitative measurement of bone turnover in the knee. Additionally, the activity concentration in a reference region, representing the ground of truth for the analysis, was calculated from the quantitative data. SPECT data from anatomical regions were also analyzed regarding the in-plane homogeneity of the uptake (homogeneity in the volume mapping to an anatomical structure).

In general, for all examined imaging protocols, we observed an underestimation of the uptake in the phantom volume typically covered by the anatomical target regions/structures. Reconstruction protocols (manufacturer specific software option “IQE”) using a data interpolation algorithm to prevent helical artifacts showed the highest underestimation. In a further optimization step, the following constraints with respect to CT-caused exposure with ionizing radiation, e.g., the lowest examined pitch (correlating to higher CT exposure) or the thin slab reconstructions (1.25 mm slice thickness), were excluded. Finally, scan protocols with a pitch of 1.375 (slice thickness 3.75 mm) in combination with one of the three reconstruction setups (Standard Plus, Bone Plus, and Bone+ Plus) provided comparable results. The median underestimation of uptake was ≤ 10.1%, with a systematic lower uptake in the volume assigned to the femur. It has to be hypothesized that the specific effect can be attributed to the structure of the load absorber system (such as different metals and polycarbonate urethane) in the X-ray beam path in this region. However, the in-slice homogeneity was good in both anatomically relevant volumes.

The reconstructed activity concentration in the reference region was overestimated in comparison to the known activity concentration. The manufacturer’s prespecified reconstruction setup underestimated the scatter in the region. In this context, it must be kept in mind that the scatter factors are estimated from point sources in homogeneous (non-active) media. This setup is not always representative of all clinical conditions (e.g., blood pool activity in the whole body or in the presence of metal implants). The scatter will be underestimated, resulting in artificially increased emission (photopeak) information. It can be hypothesized that advanced scatter corrections (e.g., based on Monte Carlo simulation or scatter estimation with deep learning) will further improve the quantitative reconstruction [27–32]. Additionally, the overestimation in the reconstructed activity concentration was influenced by an observed variation in the HU values. In our setup, we observed a slight increase in the HU values (e.g., in the reference region). As a consequence, the material density and therefore the attenuation of photons were overestimated. This effect can be attributed to deviations of the CT calibration in low-dose/ultra-low-dose imaging [33]. The effect could possibly be circumvented by using dedicated LD-CT calibration algorithms, providing quantitative correct attenuation coefficients (e.g., quantitative attenuation correction—Q. AC, GE Healthcare, which was not available for the CT used).

The propagation of the metal artifacts from CT to SPECT by CT-based attenuation correction is known for standard diagnostics and has been systematically analyzed by simulations [34] and phantom measurements [33, 35] and observed by different authors, e.g., for PET-CT [36–38]. However, discordant observations were made concerning the effect of metal implants on monitoring bone turnover. Amarasekera et al. [17] reported from a phantom validation a significantly increased (overestimated) uptake seen in the SPECT investigations in the vicinity of a specific prosthesis used in hip-resurfacing arthroplasty of the patient’s native femoral head. In contrast, our study reported a significant decrease in the measured activity concentration. It has to be hypothesized that the potential divergence was induced by differences in the geometry of the implanted system, the material composition used (covering a wide range of densities), and the examined positions (e.g., inside the cup of a rather large hip prosthesis or in some millimeters distance beside a comparable small load absorber system). These aspects potentially influence the intensity and geometry of the metal artifacts propagated to (quantitative) SPECT by the use of CT for attenuation correction. Furthermore, this discordance also illustrates the requirement for a device (e.g., extra-articular knee implant system, partial or total prosthesis) specific analysis of the metal artifacts in CT and the corresponding impact on nuclear medicine imaging (e.g., quantitative SPECT).

In diagnostic CT imaging, the usage of dedicated reconstruction algorithms, e.g., handling the artifact from the implant by specific pre-processing of the CT-raw data, represents state-of-the-art for high-end CT devices. Nevertheless, we restricted our optimization approach to standard CT reconstructions. This consideration is of importance because dedicated CT reconstruction for implants (e.g., optimized for metal artifact reduction) is currently limited to specific (high-end) SPECT-CTs of a few manufacturers. Many systems provide only standard reconstructions (filtered back projection or iterative CT reconstruction) for CT data with a wide range of specific characteristics (e.g., for reconstruction kernels) [39]. It can be hypothesized that the usage of advanced CT metal artifact reduction algorithms can further improve hybrid nuclear medicine imaging (e.g., quantification from hybrid SPECT-CT). In addition to the exclusive use for attenuation correction in quantitative SPECT, the potential application of LD-CT for delimitation of specific anatomical structures (e.g., corticalis, cancellous bone, and soft tissue) has to be examined. A potential application is the monitoring of implanted devices (e.g., localization of plates and screws, and changes in bone density). Promising results for image quality in LD-CT applications with fair detectability in specific anatomical structures (e.g., in the abdominal region) have been recently reported [40, 41].

In the actual setup, we followed a setting defined by the imperial knee protocol for CT imaging in knee replacement, resulting from an optimization examining X-ray tube voltages in the range of 100‑140 kV [42]. Nevertheless, it has to be emphasized, that besides using established scan protocols, other parametrizations (e.g., higher X-ray tube voltage) may provide the potential for further optimization in quantitative hybrid SPECT-CT imaging depending on the used CT system [43–45].

Generally, the need for an optimization of SPECT-CT imaging is illustrated by the general increase in the number of artificial joint implantations and the corresponding need for advanced imaging (e.g., loosening, metabolism, and infection).

Finally, the implant system used, in contrast to other devices that are implanted for substituting parts of a joint (or a whole joint), is designed for unloading and therefore reducing bone-turnover patterns in the knee. According to our results, the change in bone metabolism can be quantified by SPECT-CT. However, a small but significant systematic underestimation of activity concentrations in or close to the joint after implantation of the device has to be taken into account for follow-up examinations after implantation. This is of special interest for quantification in patients with a slight decrease in bone turnover (detected by quantitative SPECT-CT) after implantation of the knee unloading implant.

## Conclusion

An effect resulting from metal artifacts in the CT on quantitative SPECT imaging of bone metabolism was determined even for the specific endoprosthesis design representing a comparably small mass of dense materials. Specific knowledge of the effect is essential, especially in the interpretation of quantitative SPECT data in specific scenarios, e.g., in studying the time course of bone remodeling after unloading the knee or in decision-making before replacement of the device. In this specific translative scenario, the effect caused by the metal artifacts can cover objective measures in correlation with the baseline finding. The observed bias must be reflected critically in further studies, e.g., defining normal levels in bone metabolism after endoprosthesis surgery. Potentially, future improvements in low-dose CT reconstruction algorithms for hybrid applications can optimize quantitative SPECT imaging.

## Supplementary Information

**Additional file 1.**

## Data Availability

The datasets used and/or analyzed during the current study are available from the corresponding author on reasonable request.

## Abbreviations

3D-OSEM: 3D-Ordered Subset Expectation Maximization
CT: Computed tomography
DLP: Dose length product
LD-CT: Low-dose CT
FOV: Field of view
HU: Hounsfield units
OA: Osteoarthritis
OSEM: Ordered subset expectation maximization
ROI: Region of interest
SPECT: Single-photon emission computed tomography
U: X-ray tube voltage
