# Supplementary material for: Quantitative imaging of bone remodeling in patients with a unicompartmental joint unloading knee implant (ATLAS Knee System)—effect of metal artifacts on a SPECT-CT-based quantification
Source: EJNMMI Phys. 2021 Feb 17;8:15. doi: 10.1186/s40658-021-00360-z (PMC7889783; doi:10.1186/s40658-021-00360-z)
Supplement: Supplementary file 1 — Additional file 1. [file 40658_2021_360_MOESM1_ESM.docx]

**Supplementary Data**

**Section 1: Patients Geometry in Comparison to the used Phantom geometry**

Eight consecutive patients with unloading implants were analysed to compare the phantom set-up used with a potential patient geometry to be expected. For comparison with phantom geometry, the cross-sectional area of both knees in a single CT slice was estimated by ROI analysis. The ROIs (left/right knee) were defined by the individual skin/air interface. The analysed CT slice represented the maximum cross-sectional area for the individual patient.

Patients data:

| **No.** | **Sex** | **Age** | **Cross-sectional Area [cm^2^]** | | |
| --- | --- | --- | --- | --- | --- |
|  |  |  | **right knee** | **left knee** | **total** |
| **1** | f | 47 | 226 | 204 | 430 |
| **2** | f | 36 | 130 | 129 | 259 |
| **3** | f | 48 | 111 | 114 | 225 |
| **4** | f | 47 | 123 | 133 | 256 |
| **5** | f | 58 | 121 | 105 | 226 |
| **6** | m | 39 | 141 | 123 | 264 |
| **7** | f | 32 | 188 | 175 | 363 |
| **8** | m | 62 | 133 | 143 | 276 |

Resulting the cross-sectional area was in the average 287.4 ± 71.9 cm^2^ (median (IQR) = 261.5 (248.5/297.8) cm^2^, range 225 – 430 cm^2^). In comparison, the Jaszczak phantom used (inner diameter 20.9 cm, cylinder wall thickness of about 0.64 cm) has a cross-sectional area of 343,1 cm^2^ (active area represented by the SPECT) and 366,4 cm^2^ (area represented by the CT). The cross-sectional area of the phantom geometry slightly overestimated dimensions observed in the small cohort.

Meanwhile, the chosen geometry represented a standard in SPECT optimization and is in dimension and composition comparable to dedicated phantoms used in standard CT optimization (e.g. Catphan 500, diameter with housing = 20 cm, The Phantom Laboratory, Salem NY, USA).

**Section 2: Distribution Data and Level of Significance for Figure 5**

**Table S1:** Data distribution corresponding to the interaction plot (Figure 5A) demonstrating the effect by pitch and slice thickness on HU values.

| **Slice Thickness [mm]** | **HU Values (Pitch = 0.562)** | ***P*^*^** | **HU Values (Pitch = 1.375)** | ***P*^#^** | ***P*^+^** |
| --- | --- | --- | --- | --- | --- |
| **1.25** | 3.9 ± 47.4  3 (-13/21)  -415 - 521 | n.s. (0.14) | -15.25 ± 81.2  -4 (-39/20)  -1118 - 624 | < 0.0001 | < 0.0001 |
|  |  |  |  |  |  |
| **3.75** | 4.2 ± 28.6  3 (-7/15)  -262 - 247 |  | 1.2 ± 42.5  1 (-14/17)  -400 - 294 |  | < 0.0001 |

^*^ Significance of differences in HU values comparing slice thickness of 1.25 mm and 3.75 mm for pitch = 0.562 (Wilcoxon rank sum test)

^#^ Significance of differences in HU values comparing slice thickness of 1.25 mm and 3.75 mm for pitch = 1.375 (Wilcoxon rank sum test)

^+^ Significance of differences comparing HU values for pitch of 0.562 and 1.375 for each slice thickness (Wilcoxon rank sum test)

Data were reported by mean ± standard deviation, median (25%-/ 75%-IQR) and range.

**Table S2:** Data distribution corresponding to the interaction plot (Figure 5B) demonstrating the effect by reconstruction and X-ray tube current on HU values.

| **Reconstruction** | **HU Value** | | | ***P**** |
| --- | --- | --- | --- | --- |
|  | **I = 10 mA** | **I = 40 mA** | **I = 125 mA** |  |
| **B Plus** | 6.9 ± 50.3  7 (-24/38)  -285 - 202 | 1.63 ± 23.6  2 (-12/16)  -129 - 124 | 1.36 ± 14.7  2 (-7/124)  -103 - 72 | < 0.001^#^ |
| **B Plus IQE** | -7.0 ± 87.9  -3 (-59 50)  -455 - 366 | -1.3 ± 40.7  0 (-25/24)  -201 - 162 | 0.1 ± 24.7  1 (-7/124)  -103 - 72 | < 0.02^#^ |
| **B+ Plus** | 2.9 ± 76.7  4 (-44/51)  -400 - 294 | 0.3 ± 36.5  2 (-21/23)  -230 - 173 | 0.4 ± 22.6  1 (-12/14)  -149 - 102 | < 0.008^#^ |
| **B+ Plus IQE** | -28.3 ± 143.4  -18 (-108/64)  -1118 - 624 | -14.8 ± 70.4  -8 (-52/31)  -573 - 243 | -10.2 ± 47.8  -3 (-29/17)  -386 - 175 | < 0.002 |
| **S Plus** | 7.3 ± 31.4  7 (-11/26)  -199 - 169 | 2.0 ± 14.5  2 (-6/10)  -81 - 73 | 1.3 ± 10.0  1 (-4/7)  -85 - 60 | < 0.001 |

* Significance of differences in HU value between the examined X-ray tube currents

# Non-significant difference in HU-values from CT scans with X-ray tube current 40 mA vs. 125 mA

Note: Data were reported by mean ± standard deviation, median (25%-/ 75%-IQR) and range

**Table S3:** Data distribution corresponding to the interaction plot (Figure 5C) demonstrating the effect by reconstruction and ROI levels (e.g. ‘anatomical’ level’s femur and tibia, reference) on HU values.

| **Reconstruction** | **Tibia**  **(HU Value)** | **Femur**  **(HU Value)** | ***P**** | | **Reference^+^**  **(HU Value)** | ***P*^#^**  **(Tibia / Femur)** |
| --- | --- | --- | --- | --- | --- | --- |
| **B Plus** | 2.6 ± 32.5  2 (-12/17)  -242 – 191 | 4.2 ± 37.6  3 (-13/20)  -285 - 202 | | n.s. | 3.2 ± 29.2  2 (-10/15)  -168 - 190 | n.s. / n.s. |
| **B Plus IQE** | -5.6 ± 57.8  -2 (-29/22)  -358 – 236 | -6.0 ± 64.8  -1 (-31/25)  -455 - 366 | | n.s. | 3.4 ± 49.3  3 (-18/24)  -271 - 323 | <0.0001 / < 0.0001 |
| **B+ Plus** | -1.4 ± 50.9  1 (-24/23)  -352 – 277 | 1.7 ± 55.9  2 (-23/26)  -400 - 294 | | n.s. | 3.3 ± 44.7  2 (-16/22)  -266 - 286 | n.s. / < 0.0001 |
| **B+ Plus IQE** | -20.0 ± 92.2  -10 (-60/28)  -695 – 424 | -36.6 ± 113.7  -18 (-83/23)  -1118 - 624 | | < 0.0001 | 3.3 ± 75.9  3 (-32/37)  -424 - 486 | < 0.0001 / < 0.0001 |
| **S Plus** | 3.2 ± 19.6  2 (-6/12)  -168 – 131 | 4.6 ± 25.1  3(-8/15)  -199 - 169 | | n.s. | 2.8 ±17.4  2 (-5/9)  -125 - 155 | n.s / n.s. |

^*^ Significance of difference in HU values comparing tibia and femur ROI (Wilcoxon rank sum test)

^#^ Significance of difference in HU values comparing tibia (or femur) ROI with the corresponding reference ROI (Wilcoxon rank sum test)

^+^ Note: no significant effect by the CT reconstruction setup on HU values in reference ROI

**Table S4:** Data distribution corresponding to the interaction plot (Figure 5D) demonstrating the effect by CT pitch and reconstructed slice thickness on reconstructed activity concentration.

| **Slice [mm]** | **Activity Concentration [kBq/ml]** | | | | |
| --- | --- | --- | --- | --- | --- |
|  | **(Pitch = 0.562)** | ***P*^*^** | **(Pitch = 1.375)** | ***P*^#^** | ***P*^+^** |
| **1.25** | 194.4 ± 13.6  192 (184/206)  163 - 228 | n.s. | 191.4 ± 15.6  189 (180/205)  141-205 | < 0.0001 | <0.0001 |
|  |  |  |  |  |  |
| **3.75** | 195.6 ± 12.9  193 (185/206)  167 - 228 |  | 194.9 ± 13.1  193 (184/206)  163 - 228 |  | n.s. |

^*^ Significance of differences in activity concentrations reconstructed using CT data with slice thickness of 1.25 mm and 3.75 mm (pitch = 0.562) for attenuation correction (Wilcoxon rank sum test).

^#^ Significance of differences in activity concentrations reconstructed using CT data with slice thickness of 1.25 mm and 3.75 mm (pitch = 1.375) for attenuation correction (Wilcoxon rank sum test).

^+^ Significance of differences in activity concentrations using CT data for attenuation correction of 0.562 and 1.375 for each slice thickness (Wilcoxon rank sum test).

**Table S5:** Data distribution corresponding to the interaction plot (Figure 5E) demonstrating the effect by X-ray tube current on reconstructed activity concentration for different reconstruction setups.

| **Reconstruction** | **Reconstructed Activity Concentration [kBq/ml]** | | |  |
| --- | --- | --- | --- | --- |
|  | **I = 10 mA** | **I = 40 mA** | **I = 125 mA** | ***P**** |
| **B Plus** | 195.7 ± 13.2  193 (185/207)  166 – 228 | 195.2 ± 12.9  193 (185/206)  166 - 227 | 195.0 ± 12.8  193 (185/206)  166 - 227 | n.s. |
|  |  |  |  |  |
| **B Plus IQE** | 192.7 ± 14.8  190 (181/206)  156 - 228 | 193.5 ± 14.0  191 (183/206)  159 - 227 | 193.5 ± 13.7  191 (183/205)  161 - 227 | n.s. |
|  |  |  |  |  |
| **B+ Plus** | 195.2 ± 13.5  193 (185/207)  163 - 228 | 194.9 ± 13.2  193 (184/206)  163 - 227 | 194.8 ± 13.0  193 (184/206)  165 – 227 | n.s. |
|  |  |  |  |  |
| **B+ Plus IQE** | 189.5 ± 17.6  188 (177/205)  141 - 228 | 191.6 ± 15.6  190 (180/205)  147 - 227 | 192.0 ± 15.1  190 (181/205)  150 - 227 | 0.003^+^ |
|  |  |  |  |  |
| **S Plus** | 195.4 ± 13.3  193 (185/207)  165 - 228 | 194.7 ± 13.1  192 (184/206)  164 - 227 | 194.4 ± 13.1  192 (184/205)  164 - 227 | n.s. |

* significance of effects by X-ray tube current for different examined reconstruction setups

^+^ non-significant difference in reconstructed activity concentration for 40 mA vs. 120 mA

**Table S6:** Data distribution corresponding to the interaction plot (Figure 5F) demonstrating the effect by reconstruction and ROI levels (e.g. ‘anatomical’ level’s femur and tibia, reference) on reconstructed activity concentration.

| **Reconstruction** | | **Region** | | | | |  | |
| --- | --- | --- | --- | --- | --- | --- | --- | --- |
|  | **Tibia** | | **Femur** | ***P**** | **Reference ^+^** | ***P*^#^ (tibia / femur)** | |  |
| **B Plus** | 188.1 ± 9.6  188 (182/198)  168 - 222 | | 190.4 ± 11.0  186 (182/191)  166 - 220 | < 0.0001 | 207.3 ± 8.5  208 (203/213)  184 - 228 | < 0.0001 (both) | |  |
| **B Plus IQE** | 186.0 ± 9.8  185 (180/190)  158 - 218 | | 186.6 ± 12.1  185 (178/195)  156 - 222 | n.s. | 207.1 ± 8.5  208 (203/212)  184 - 228 | < 0.0001 (both) | |  |
| **B+ Plus** | 187.8 ± 9.8  187 (181/197)  165 - 222 | | 189.9 ± 11.3  186 (182/191)  164 - 220 | < 0.0001 | 207.4 ± 8.5  208 (203/213)  184 - 228 | < 0.0001 (both) | |  |
| **B+ Plus IQE** | 183.7 ± 11.0  184 (177/189)  148 - 218 | | 182.2 ± 14.0  181 (173/191)  141 - 221 | 0.0005 | 207.2 ± 8.5  208 (184/212)  184 – 228) | < 0.0001 (both) | |  |
| **S Plus** | 187.7 ± 9.4  189 (182/198)  168 - 222 | | 189.5 ± 11.3  186 (183/191)  167 - 220 | < 0.0001 | 207.3 ± 8.5  208 (203/213)  183 - 228 | < 0.0001 (both) | |  |

^*^ Significance of difference in reconstructed activity concentration comparing tibia and femur ROI (Wilcoxon rank sum test)

^#^ Significance of difference in reconstructed activity concentration comparing tibia (or femur) ROI with the corresponding reference ROI (Wilcoxon rank sum test)

^+^ Note: no significant effect by reconstruction setup on activity concentration in reference ROI

**Section 3: Evaluation of a patient examination for illustrating the effect of the CT reconstruction setup on the quantitative SPECT data.**

Data obtained from a female patient (48 yr.) with osteoarthritis of the right knee and implanted Atlas^®^ Knee System were analysed. The corresponding CT data (120 kV, 55 mA, trot = 0.8 s, pitch = 1.375) were reconstructed by using filter setup ‘Std Plus’. The activity concentration in the medial knee joint was analysed in three consecutive slices. Furthermore, a set of three consecutive ROIs was placed in the right femur shaft representing a reference region for evaluation of uptake outside of volumes affected by metal artefact from unloading implant. The set was positioned about 10 cm cranial of the knee. ROIs were drawn by an observer experienced in SPECT/CT imaging. The identic ROI sets were used for each reconstruction.

**Figure S1:** Accumulation of ^99m^Tc-2,3-dicarboxypropane-1,1-diphosphonate (^99m^Tc-DPD, TECEOS^®^, IBA Molecular, CIS Bio GmbH, Berlin, Germany) in medial compartment of the right femur is shown in (A) sagittal, (B) coronal and (C) axial slices. The level of the target region (medial knee joint) and of a reference region (femur shaft) analysed for methodological comparison were indicated.

**Figure S2:**

Effects from CT reconstruction on activity concentration were analysed for (**A**) the target volume (medial knee joint) and (**B**) a reference region (femur shaft) outside the volume affected by metal artefact. Significances and activity concentration were also documented by Table S7.

**Table S7:** Effect by CT reconstruction on reconstructed activity concentration in the medial knee joint.

| **Reconstruction** | **Activity concentration**  **[kBq/ml]** | ***P*^#^** |
| --- | --- | --- |
| B Plus | 216.9 +/- 78.8  217.0 (163 – 268.0)  58.0 – 387. | 0.044 |
| B PlusIQE | 206.9 +/- 78.0  202.4 (50.4/259.1)  50.4 – 376.4 | n.s. |
| B+ Plus | 216.4 +/- 78.5  215.0 (162.2 – 268.0)  59.0 - 387.0 | (0.053) |
| B+ Plus IQE | 186.9 +/- 76.5  179.1 (134.3 – 239.8)  33.5 - 355.5 | - |
| Std Plus | 217.2 +/- 78.9  218.0 (163.0 – 268.0)  58.0 - 387.0 | 0.041 |

# significance of differences in activity concentration in comparison to reconstruction setup ‘B+ PlusIQE’
